# Significance of ZEB2 in the immune microenvironment of colon cancer

**DOI:** 10.3389/fgene.2022.995333

**Published:** 2022-08-22

**Authors:** Hao Xie, Zhaoying Wu, Zhenhan Li, Yong Huang, Junwei Zou, Hailang Zhou

**Affiliations:** ^1^ Department of Gastrointestinal Surgery, The Second Affiliated Hospital of Wannan Medical College, Wuhu, China; ^2^ School of Clinical Medicine, Wannan Medical College, Wuhu, China; ^3^ Department of Gastroenterology, Lianshui People’s Hospital Affiliated to Kangda College of Nanjing Medical University, Huai’an, Jiangsu, China

**Keywords:** ZEB2, immune infiltration, methylation, multi-omics, COAD

## Abstract

**Background:** ZEB2 is a protein-coding gene that is differentially expressed in tumors and can regulate the growth of tumor cells. This study investigated the specific regulatory mechanism of ZEB2 in COAD, a common cancer with high rates of morbidity and mortality.

**Methods:** Multi-omics panoramic display of expression and function of ZEB2 in colon cancer. R software was used to study the expression of ZEB2 in 33 types of cancer. Furthermore, RT-PCR was used to detect the expression of ZEB2 in colon cancers and para-cancer tissues, as well as in colon cancer cells and normal cells. The ssGSEA was then used to explore the relationship between ZEB2 and immune cells, with UALCAN, EWAS and MEXPRESS applied to explore the methylation of ZEB2. The relationship between immunomodulators and chemokines (or receptors) based on expression data, copy number data, methylation data, and mutation data of ZEB2 was investigated using TISIDB. Finally, a protein interaction network of ZEB2 was constructed, and GO and KEGG analyses were performed on the differentially expressed genes.

**Results:** ZEB2 is downregulated in most cancers, including COAD. The infiltration of the immune cells NK CD56 and Th17 cells was negatively correlated with ZEB2 expression, while the other 22 cells were positively correlated with ZEB2 expression. The DNA methylation of ZEB2 and the methylation of the ZEB2 protein on the EWAS website increased significantly. Analysis of the methylation levels and ZEB2 expression revealed that only the DNA methylation level and the expression of ZEB2 were significantly negatively correlated. The tumor-infiltrating lymphocytes positively correlated with the expression of ZEB2 but negatively correlated with the methylation of ZEB2. The same trend was observed for immunomodulators, chemokines, and receptors. The network showed that the protein performed certain biological functions, thereby affecting disease symptoms.

**Conclusion:** These findings provide evidence that ZEB2-based therapy may represent a powerful treatment strategy for COAD.

## Introduction

COAD is currently the third most widespread cancer in the world and the fourth most deadly cancer, with increasing rates of morbidity and mortality (Sung et al., 2021). Colon cancer may not be easily detected in the early stages, and in many cases, there are no symptoms until later stages, hence the high fatality rate (Arnold et al., 2017). Most colon cancers are caused by polyps which later develop lesions and eventually develop into colon cancer. The continuous accumulation of genetic and epigenetic changes lead to the inactivation of tumor suppressor genes, the activation of oncogenes, and ultimately the formation and maintenance of tumors (Guo et al., 2019). There is an urgent need to investigate potential therapeutic targets for COAD progression.

The ZEB2 gene codes for a member of the Zfh-1 family of two-handed zinc finger/homeodomain proteins (Scott and Omilusik, 2019). It is located in the nucleus and functions as a DNA-binding transcriptional repressor that interacts with activated SMADs. Mutations in this gene are associated with Hirschsprung disease/Mowat-Wilson syndrome (Hegarty et al., 2015), and alternatively spliced transcript variants have been found for this gene. The biological functions of ZEB2 include a transcription inhibitor that can bind to the DNA in different promoters (Long et al., 2005; Chen et al., 2010), an inhibitor of E-cadherin transcription, and MEOX2 expression. ZEB2 is differentially expressed in tumors and can regulate the growth of tumor cells (Fardi et al., 2019) but the specific role of ZEB2 in colon cancer has not been explored in depth.

The TCGA-COAD dataset was used in this study to conduct immune infiltration, methylation, and enrichment analyses, focusing on the mechanism of ZEB2 in COAD to analyze its clinical significance.

## Materials and methods

### Data collection

Thirty-three types of cancers from TCGA were analyzed and the downloaded TPM format of TCGA and GTEx was processed uniformly by the Toil (Vivian et al., 2017) process from UCSC Xena (https://xenabrowser.net/datapages/) RNAseq data (Liu et al., 2021a). Since the number of adjacent tissue samples corresponding to the tumor in TCGA is relatively small, normal tissues were added to the GTEx dataset as a control for further evaluation. After log2 conversion of the RNAseq data, the expression was compared between samples. There was a total of 15,776 unpaired samples, comprising 4,683 normal samples and 11,093 tumor samples; the paired samples totaled 11,093. ROC curve is used to diagnose the stability of gene prediction (Liu et al., 2021c; Li et al., 2022). The R software (version 3.6.3) and ggplot2 were used for statistical analysis and visualization, ns *p* ≥ 0.05, *p < 0.05, **p < 0.01, ***p < 0.001.

### Cell culture

The Suzhou Medical University (Suzhou, China) provided the following human colon cancer cell lines Caco-2, HT29, SW480, and HCT116, as well as the normal colon cell line HIEC. There were cultured in DMEM (HyClone, United States) supplemented with 10% FBS (Gibco, United States) and 100 μg/ml streptomycin/penicillin (Hyclone) in a 5% CO_2_ humidified environment at 37°C. The cells were passaged every 2–3 days using 0.25% trypsin (Hyclone).

### Clinical sample collection

Six colon cancer tissues and matched adjacent normal tissues were collected from COAD patients of the Second Affiliated Hospital of Wannan Medical College. Among these patients, none of them had been subjected to radiotherapy, chemotherapy, or immunotherapy. The Ethics Committee of the Second Affiliated Hospital of Wannan Medical College granted permission to perform the research and every participant provided informed consent.

### RT-PCR

The expression of ZEB2 in tumor and normal tissues was compared by qRT-PCR. The expression of ZEB2 in different colon cancer cell lines was also quantified by qRT-PCR, with at least three biological replicates per sample. The total RNA concentration of each sample was adjusted to be the same before reverse transcription using the ChamQ Universal SYBR qPCR Master Mix and HiScript II Q RT SuperMix for qPCR (Nanjing Novozan Biotechnology Co. ltd.). The relative mRNA expression was calculated by the 2^-ΔΔCT^ method and normalized to the internal reference GAPDH. The primer sequences were as follows: ZEB2: Forward: 5′-AAA​ACC​TCG​CCA​AGA​GTG​TC-3′, Reverse: 5′-GAG​GCG​TAA​CAC​GTC​AGT​CC-3′, GAPDH: Forward: 5′-GAA​GGT​GAA​GGT​CGG​AGT​C-3′, Reverse: 5′-GAA​GAT​GGT​GAT​GGG​ATT​TCC-3′.

### Immune infiltration

The immune infiltration algorithm used was ssGSEA (Hanzelmann et al., 2013) and the data from TCGA (https://portal.gdc.cancer.gov/) was COAD project FPKM format RNAseq data (Liu et al., 2021a). First, the RNAseq data in FPKM format were converted into TPM format, and then a log2 conversion was performed (Liu et al., 2021b). The markers of 24 immune cells were taken from an article of Immunity (Bindea et al., 2013; Han et al., 2021).

### Methylation of zinc finger E-box-binding homeobox 2

UALCAN (http://ualcan.path.uab.edu/) and EWAS(http://www.ewas.org.cn) are comprehensive and convenient visualization websites (Chandrashekar et al., 2017). Seventy-one methylation probes related to ZEB2 were obtained and subjected to enrichment analysis. MEXPRESS is a convenient website that can visually display the relationship between different independent variables, such as gene DNA methylation level, expression level, and clinical data (https://mexpress.be/) (Koch et al., 2019).

### TISIDB

Tumor and immune system interaction database (TISIDB) is a web portal for tumor and immune system interactions (http://cis.hku.hk/TISIDB/) (Ru et al., 2019) that details the relationships between the abundance of tumor-infiltrating lymphocytes (TILs) based on the expression, copy number, methylation and mutation data of ZEB2. The relative abundance of TILs was inferred by using GSVA based on the gene expression profile.

### Construction of the protein interaction network of zinc finger E-box-binding homeobox 2

STRING (https://cn.string-db.org/) is the most comprehensive large-scale protein interaction database (Szklarczyk et al., 2021). Ten proteins most closely related to the function of ZEB2 were obtained. GeneMANIA was used to generate hypotheses about gene functions, analyze the gene lists and determine the priority of genes for functional analysis (Warde-Farley et al., 2010).

### Enrichment analysis

To further explore the role of ZEB2 in tumors, GO/KEGG enrichment analysis (Yu et al., 2012) and single-gene difference analysis (Love et al., 2014) were performed to reveal its potential biological functions. The target molecule is ZEB2, the low expression group is 0%–50%, and the high expression group is 50%–100%. Gene set enrichment analysis (GSEA) was also conducted using c2.cp.v7.2.symbols.gmt. Generally, the threshold for significant enrichment is considered to be a false discovery rate (FDR) of <0.25 and p.adjust <0.05 (Xu et al., 2021).

## Results

### Zinc finger E-box-binding homeobox 2 is differentially expressed in different tumors

**FIGURE 1:**
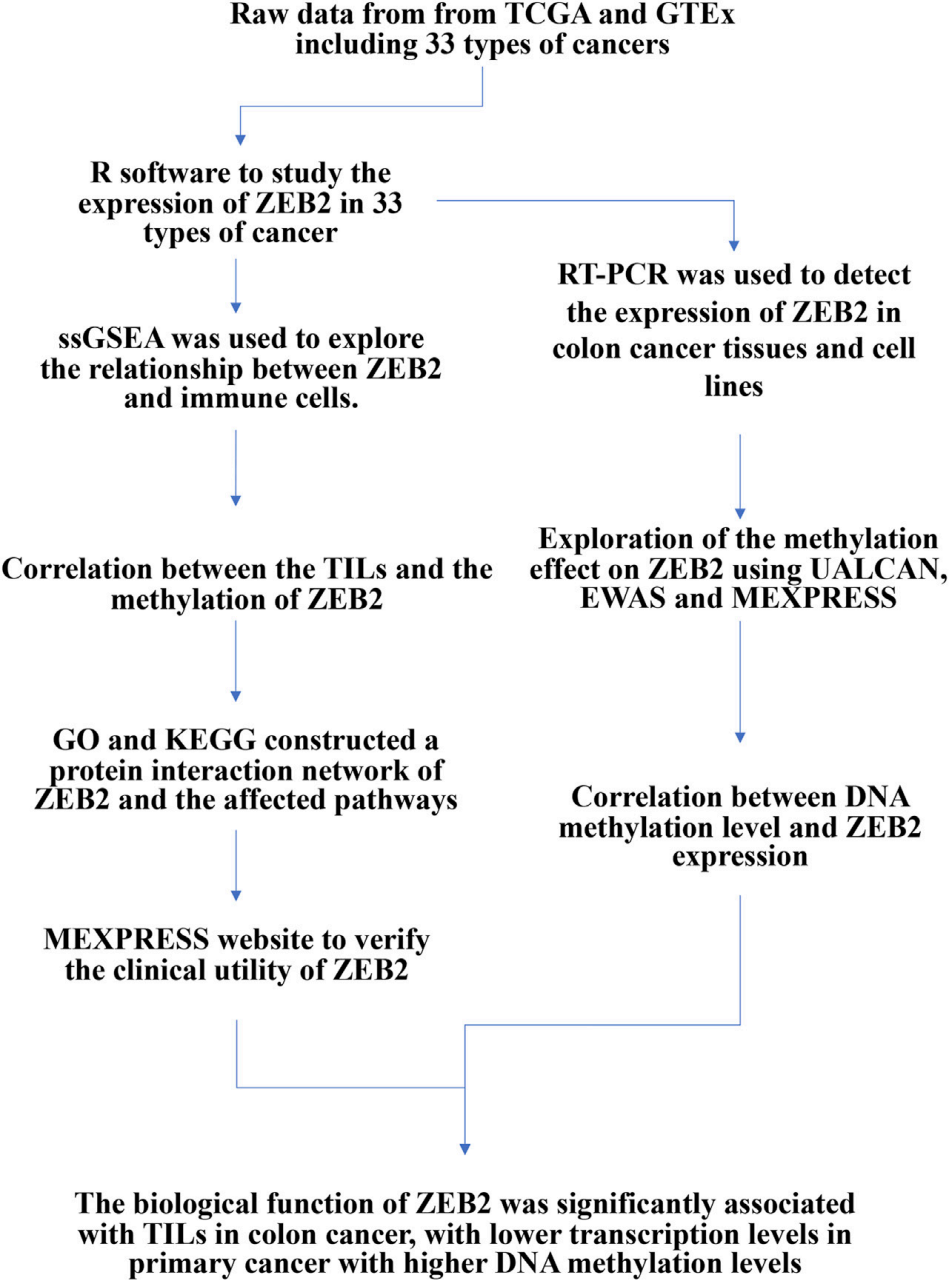
Study flow chart.

**FIGURE 2:**
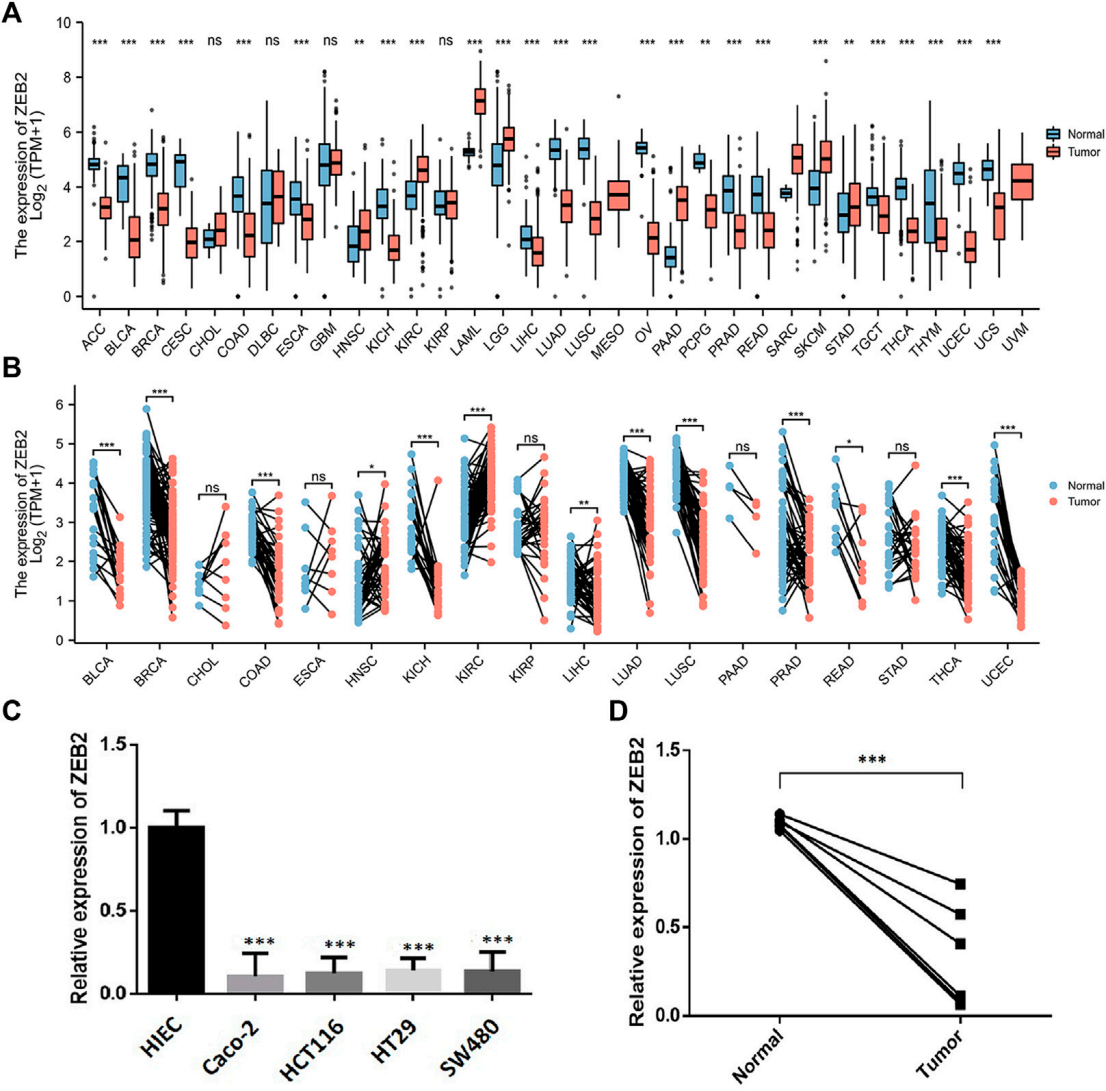
ZEB2 expression in different cancers. **(A)** ZEB2 expression in unpaired samples of different cancers. **(B)** ZEB2 expression in paired samples of different cancers. **(C)** Difference in the ZEB2 transcription between normal colon cells and colon cancer cell lines. **(D)** Difference of ZEB2 transcription between normal tissues and colon cancer tissues.

The study flow chart is provided in Figure 1. The unpaired samples revealed that ZEB2 is differentially expressed in several tumors and downregulated in most tumors, with only CHOL, DLBC, GBM, and KIRP showing no expression differences (Figure 2A). In the samples with matched data pairs, it was also found that ZEB2 was downregulated in many cancers but there was no difference in expression in ESCA, PAAD, and STAD (Figure 2B). The transcription level of ZEB2 was compared in normal tissues and colon cancer by qPCR and also verified in cancer cell lines and normal cells (Figures 2C,D). In addition, there was a significantly low expression of ZEB2 in COAD.

### Immune infiltration

**FIGURE 3:**
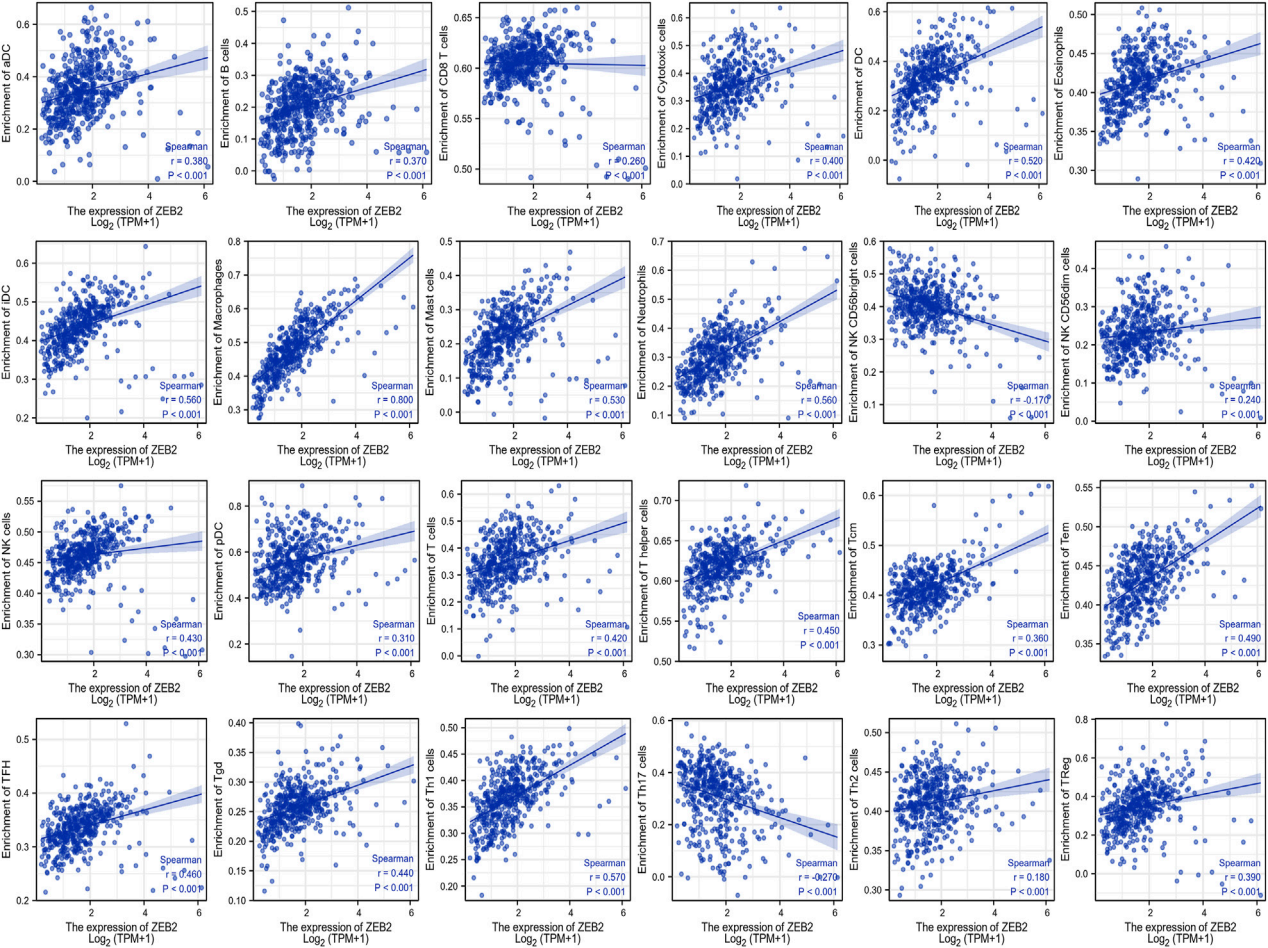
The correlation between ZEB2 expression and 24 immune cells based on TCGA data.

**FIGURE 4:**
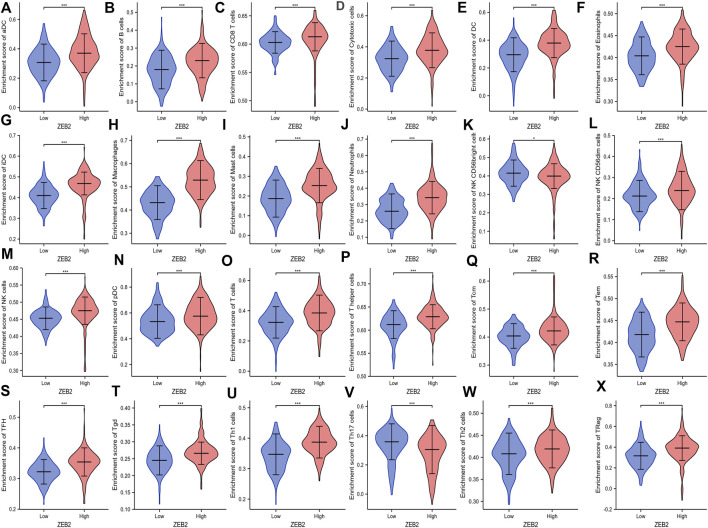
**(A–X)** Enrichment of 24 types of immune cells in the ZEB2 high- and low-expression groups.

The correlation between ZEB2 and 24 immune cells was analyzed based on the data from TCGA, with only NK CD56 bright cells and Th17 cells being negatively correlated with ZEB2 expression. The remaining 22 immune cells were all positively correlated with ZEB2 expression (Figure 3). The group comparison chart showed that the immune infiltration score of the ZEB2 high-expression group was significantly higher than that of the low-expression group, and the remaining two immune cells were significantly decreased (Figure 4). The expression of ZEB2 and the level of immune cell infiltration were clearly illustrated and confirmed in the follow-up scatter plot. As the expression level of ZEB2 increases, the level of immune infiltration of the 22 immune cells also significantly increases, while the other two types of cells decreased (Supplementary Figure S1).

### Methylation of zinc finger E-box-binding homeobox 2

**FIGURE 5:**
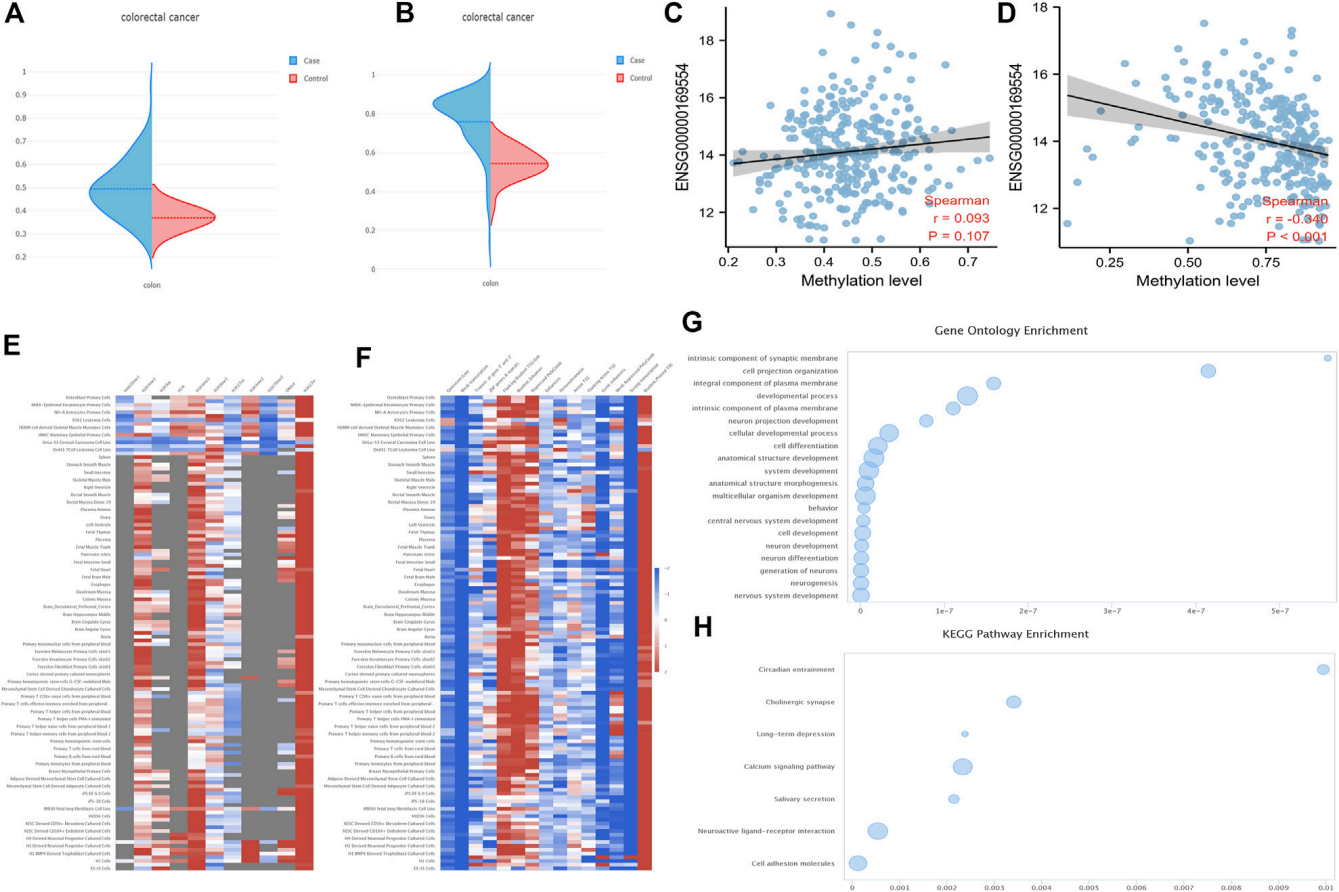
The DNA methylation levels of ZEB2. **(A)** The DNA methylation level of the ZEB2 body. **(B)** Methylation level of the ZEB2 promoter on the EWAS website. **(C)** The correlation between DNA methylation of the body and ZEB2 expression. **(D)** The correlation between DNA methylation of the promoter and ZEB2 expression. **(E)** The different methylation forms and the enrichment of various histones in different tissues and cells. **(F)** The chromatin state enrichment results of the ZEB2 methylation probe. **(G,H)** The GO/KEGG enrichment analysis of ZEB2 methylation probes.

**FIGURE 6:**
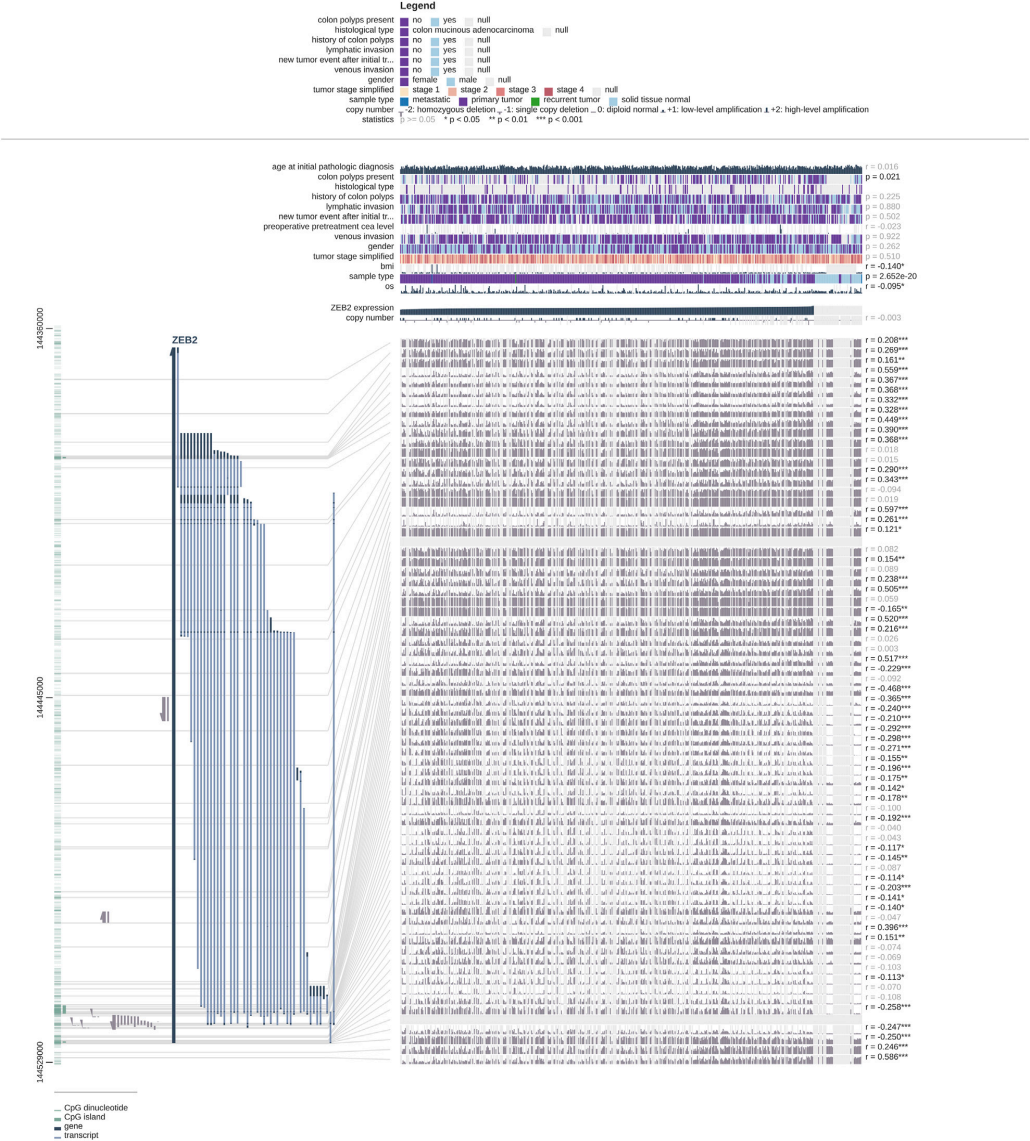
Correlation between clinical features and ZEB2 methylation probes.

Firstly, the DNA methylation levels of ZEB2 in tumors and normal tissues were obtained from the UALCAN website (Supplementary Figure S2A; Normal-vs.-Primary 3E-07). The 34 probes were all drawn with scatter plots, showing that most probes were negatively correlated with the expression level, with a few probes showing no correlation or a positive correlation (Supplementary Figure S2). In addition, the DNA methylation levels of both the ZEB2 body and the ZEB2 promoter on the EWAS website increased significantly (Figures 5A,B), with only the relationship between the DNA methylation of the promoter and the expression of ZEB2 being significantly negatively correlated (Figures 5C,D). The different methylation forms and the enrichment of various histones in different tissues and cells were presented in the form of a heat map (Figure 5E) showing the histone enrichment of H3K4me1, H3K4me3, H3K9me3, and H3K27me3 in the colonic mucosa. The heat map of the chromatin state enrichment results of the ZEB2 methylation probe showed that flanking bivalent TSS/Enh increased significantly (Figure 5F). The GO/KEGG enrichment analysis on the ZEB2 methylation probes (Figures 5G,H) showed that ZEB2 may have certain biological functions, such as cell projection organization and is an integral component of the synaptic membrane and the plasma membrane, and may affect the cholinergic synapse, calcium signaling pathway, salivary secretion, neuroactive ligand-receptor interaction, cell adhesion molecules, and other pathways. The MEXPRESS website was used to explore the correlation between OS prognosis, sample type, BMI, and other variables and ZEB2 methylation probes (Figure 6), revealing that both OS and BMI are negatively correlated with expression levels and have a strong correlation with many methylation probes.

### The relationship between zinc finger E-box-binding homeobox 2 methylation level and immune infiltration

**FIGURE 7:**
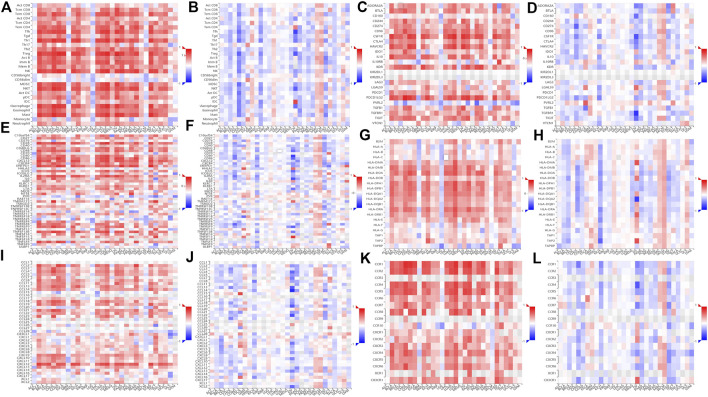
The relationship between the methylation level of ZEB2 and immune infiltration. **(A)** The correlation between TILs and ZEB2 expression. **(B)** The correlation between TILs and ZEB2 methylation. **(C)** The correlation between immunosuppressants and ZEB2 expression. **(D)** The correlation between immunosuppressants and ZEB2 methylation. **(E)** The correlation between immune activators and ZEB2 expression. **(F)** The correlation between immune activators and ZEB2 methylation. **(G)** The correlation between MHC molecules and ZEB2 expression. **(H)** The correlation between MHC molecules and ZEB2 methylation. **(I)** The correlation between chemokines and ZEB2 expression. **(J)** The correlation between chemokines and ZEB2 methylation. **(K)** The correlation between receptors and ZEB2 expression. **(L)**: The correlation between receptors and ZEB2 methylation.

The TISIDB website was used to conduct a comparison of TILs, three kinds of immunomodulators, and chemokines (or receptors) in terms of their correlation with expression levels and methylation levels of ZEB2. Most TILs were positively correlated with the ZEB2 expression but negatively correlated with ZEB2 methylation (Figures 7A,B). In addition, most immunomodulators, immunosuppressants (Figures 7C,D), immune activators (Figures 7E,F), or MHC molecules (Figures 7G,H), were also positively correlated with expression levels and negatively correlated with the degree of methylation. The same trend was observed for chemokines (Figures 7I,J) and receptors (Figures 7K,L). Taken together, as the level of ZEB2 DNA methylation increases, the expression of ZEB2 decreases significantly, with the level of immune infiltration also decreasing significantly, thereby promoting the occurrence and development of tumors.

### Construction of the protein interaction network of zinc finger E-box-binding homeobox 2

**FIGURE 8:**
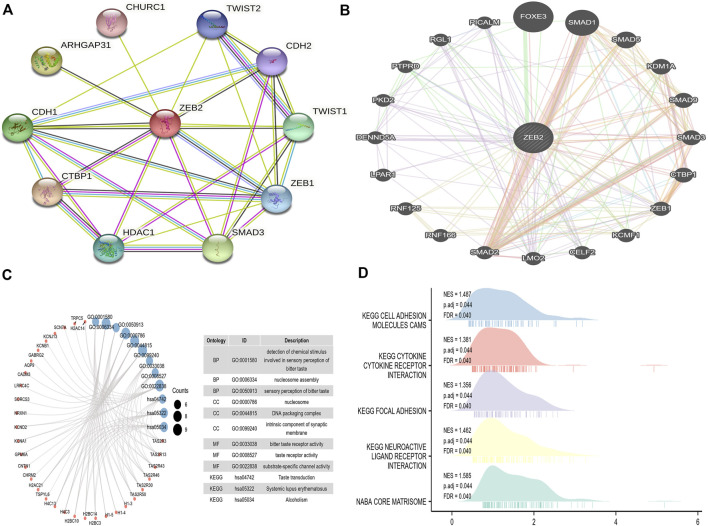
The protein interaction network and enrichment analysis of ZEB2. **(A)** The PPI network on the STRING website. **(B)** The PPI network constructed on the GeneMANIA website. **(C)** The GO/KEGG analysis of different genes. **(D)** The GSEA analysis of ZEB2.

A PPI network comprising 11 points and 31 edges (PPI enrichment *p*-value was 1.7e-05) (Figure 8A) was built using the STRING website. From the PPI network constructed in the GeneMANIA database, it was observed that physical interactions accounted for 77.64% of the network, co-expression accounted for 8.01%, predicted accounted for 5.37%, co-localization accounted for only 3.63%, genetic interactions accounted for 2.87%, pathway accounted for 1.88%, and shared protein domains only accounted for 0.60% (Figure 8B). This indicates that most proteins in this network may interact directly to perform biological functions, thereby affecting disease symptoms.

### Enrichment analysis of zinc finger E-box-binding homeobox 2

The TCGA data were used for single-gene difference analysis. The total number of gene IDs was 56,493, among which there were different types of molecules, such as protein-coding, lncRNA, and miRNA. There were 9,191 IDs falling within the threshold of |log2(FC)| > 1 and p.adj < 0.05. Under this threshold, the number of high-expression genes (logFC is positive) in the high group was 8,667, and the number of low-expression genes (logFC is negative) in the low group was 524. The condition |log2(FC)| > 2.5 and p.adj < 0.05 obtained a total of 137 encoded proteins, which were used for ordinary GO/KEGG enrichment analysis. Under the conditions of p.adj < 0.1 and qvalue < 0.2, BP had 29 entries, CC had 38 entries, and both MF and KEGG have six entries (Figure 8C). The logFC in the previous single-gene difference analysis was used as the molecular ranking to evaluate whether the gene set was significant, obtaining a total of 55 data sets satisfying FDR (q value) < 0.25 and p.adj < 0.05 (Figure 8D, Supplementary Figure 3).

## Discussion

This study explored the specific expression of ZEB2 in 33 human cancers and found there to be significant differences between several tumors. For example, ZEB2 expression decreased significantly in tumors such as ACC, BLCA, BRCA, and COAD but significantly increased in HNSC, LAML, LGG, PAAD, and SKCM.

According to the literature, ZEB2 achieves RNA polymerase II cis-regulatory region sequence-specific DNA binding (Gaudet et al., 2011) and exhibits DNA-binding transcriptional repressor activity. As a regulator of epithelial-mesenchymal transition, ZEB2 is strongly downregulated by miR-221 (Chen et al., 2010), and ZEB2 expression was increased, which in turn inhibited the expression of G9A and MTA3 by recruiting G9A/NuRD (MTA1), thereby promoting the metastasis and progression of breast cancer (Si et al., 2015).

It is well known that immune cells play a very important role in the tumor microenvironment (Galluzzi et al., 2020). After processing and visualizing the immune infiltration data in COAD, we found that the expression of ZEB2 was strongly correlated with 24 types of immune cells, with 22 of those immune cells showing a strong positive correlation. Combined with the expression of ZEB2 in COAD, this indicates that the patient’s immune system may be significantly suppressed in COAD, allowing the development of a microenvironment that promotes tumorigenesis and tumor development. As current tumor immunotherapy mainly targets lymphocytes, the reduced lymphocyte infiltration of COAD would dampen the effect of immunotherapy. Therefore, research on the microenvironment in COAD can help to overcome immune suppression and increase the response of tumors to immunotherapies (Qi et al., 2022).

DNA methylation is an epigenetic mechanism that is deregulated in precancerous colon lesions (Petko et al., 2005). Methylation is a form of DNA chemical modification that can alter gene expression without changing the DNA sequence (Hashimoto et al., 2010) and may promote tumor progression by inhibiting the plasticity of cell differentiation (Klutstein et al., 2016). The DNA methylation level of ZEB2 was significantly increased, that is, negatively correlated with ZEB2 expression. The deregulation of DNA methylation is an epigenetic alteration that takes place during colon carcinogenesis and can be detected from precancerous lesions, such as ACP, to advanced stages of COAD. At the same time, there was no correlation with protein methylation, which means that it affects the expression of ZEB2 epigenetically. Various increases in methylation have been found in colon cancer tissues (Jung et al., 2020). H3K4me1, H3K4me4, H3K4me3, etc. are all increased significantly (Akhtar-Zaidi et al., 2012; Triff et al., 2017). Among these, H3K4me4 methylation enrichment is usually considered to occur in the active position of transcription factors, so the transcription repressor stimulated this function causing the expression of ZEB2 to decrease significantly. In addition, ZEB2 positively correlated with m6A methylases and the demethylase FTO showed a significant positive correlation with ZEB2 (Supplementary Figures 4A,B). Deregulation of epigenetic mechanisms has relevant effects on the development and progression of COAD and may have clinical utility as a tumor biomarker of COAD (Du et al., 2020).

The PPI network showed that ZEB2 had energy interactions with multiple genes. The specific internal connection between ZEB2 and interacting genes may be crucial to its role in tumor progression. Multiple genes may interact to regulate tumor progression. Further functional enrichment analysis showed that ZEB2 and its interacting genes are mainly involved in cholinergic synapses, calcium signaling pathways, saliva secretion, neuroactive ligand-receptor interactions, cell adhesion molecules, and other pathways.

The MEXPRESS website was used to analyze the relationship between ZEB2 expression and other factors, with OS and BMI showing a negative correlation. The occurrence of colon cancer is closely related to colon polyps and as the expression of ZEB2 decreases, so the possibility of colon polyps occurring also increases. We can also infer that the expression of ZEB2 is related to the incidence of colon cancer, so the decreased expression of ZEB2 may promote the occurrence of colon cancer.

Immunotherapy has had significant success in cancer treatment in recent years (Kennedy and Salama, 2020). Regarding the relationship between the level of ZEB2 methylation and immune infiltration, most immunomodulators were also positively correlated with the expression level, with ZEB2 expression and methylation negatively correlated with the abundance of most TILs (Zhu et al., 2021).

A ROC curve was constructed based on the TCGA data to explore the clinical significance of ZEB2 showing that ZEB2 exhibited excellent diagnostic accuracy (AUC = 0.844). The risk factor diagram showed that with a significant increase in risk score, the prognosis of patients would become worse (Supplementary Figures S4C,D, S5). The baseline data table was also constructed to describe the basic situation of each study subject, and the combination subgroup can be used to evaluate whether the constituent ratios of different clinical variables are different among the subgroups (Supplementary Table S1). In addition, a proportional risk regression model (a univariate and multivariate Cox regression) was constructed with survival outcome and survival time as dependent variables to analyze the effects of many independent variables on survival (Supplementary Table S2).

In conclusion, our findings showed that ZEB2 was decreased in COAD and strongly correlated with 24 different types of immune cells, therefore ZEB2 may be a potential therapeutic target for colon cancer.

## Data Availability

The datasets presented in this study can be found in online repositories. The names of the repository/repositories and accession number(s) can be found in the article/Supplementary Material.

## Abbreviations

COAD, Colon adenocarcinoma; ZEB2, Zinc Finger E-Box-Binding Homeobox 2; TCGA, The Cancer Genome Atlas; TPM, Transcripts per million reads; GTEx, Genotype-Tissue Expression; FBS, Foetal bovine serum; ssGSEA, The built-in algorithm of the GSVA package; FPKM, Fragments per kilobase per million; TILs, Tumor-infiltrating lymphocytes; GSVA, Gene set variation analysis; GSEA, Gene set enrichment analysis; FDR, False discovery rate; CC, Cellular component, MF, Molecular function.
