# Supplementary figures and images for: Significance of ZEB2 in the immune microenvironment of colon cancer
Source: Front Genet. 2022 Aug 22;13:995333. doi: 10.3389/fgene.2022.995333 (PMC9442042; doi:10.3389/fgene.2022.995333)

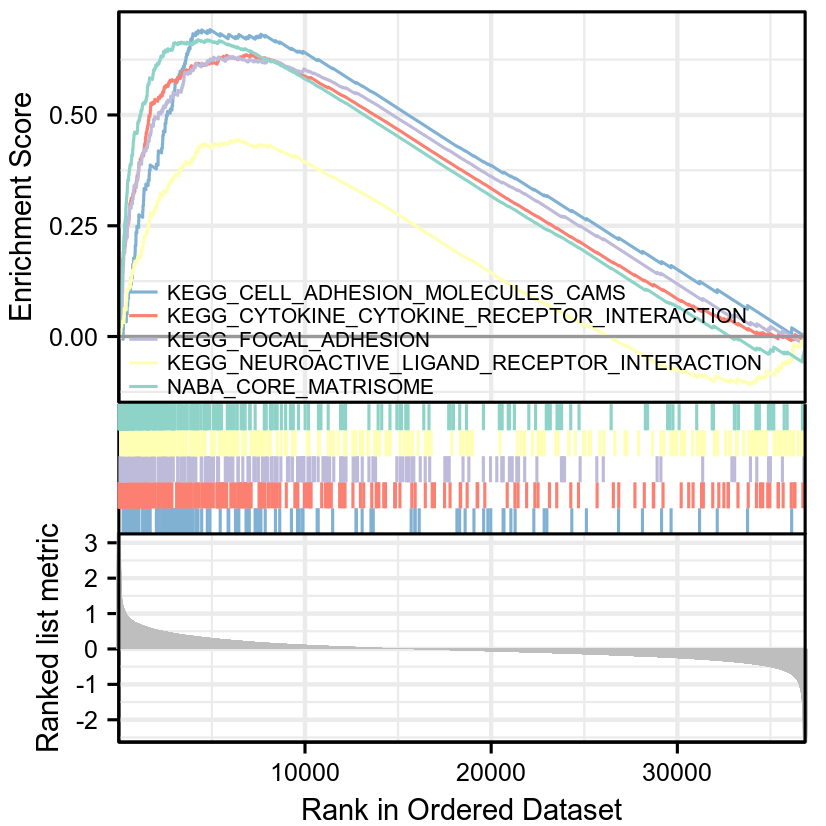

Supplement: Supplementary file 1 [file Image3.TIFF]

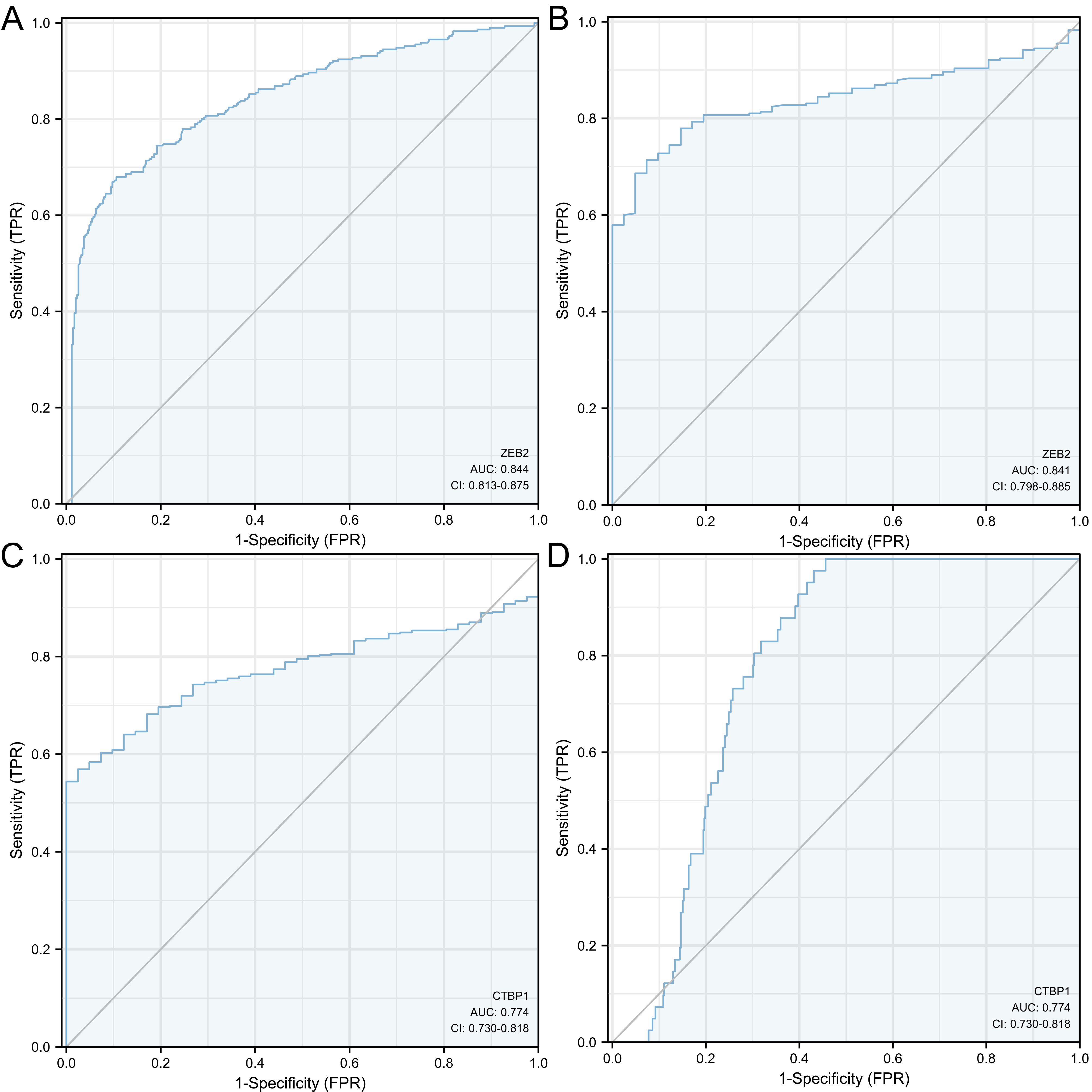

Supplement: Supplementary file 3 [file Image5.TIFF]

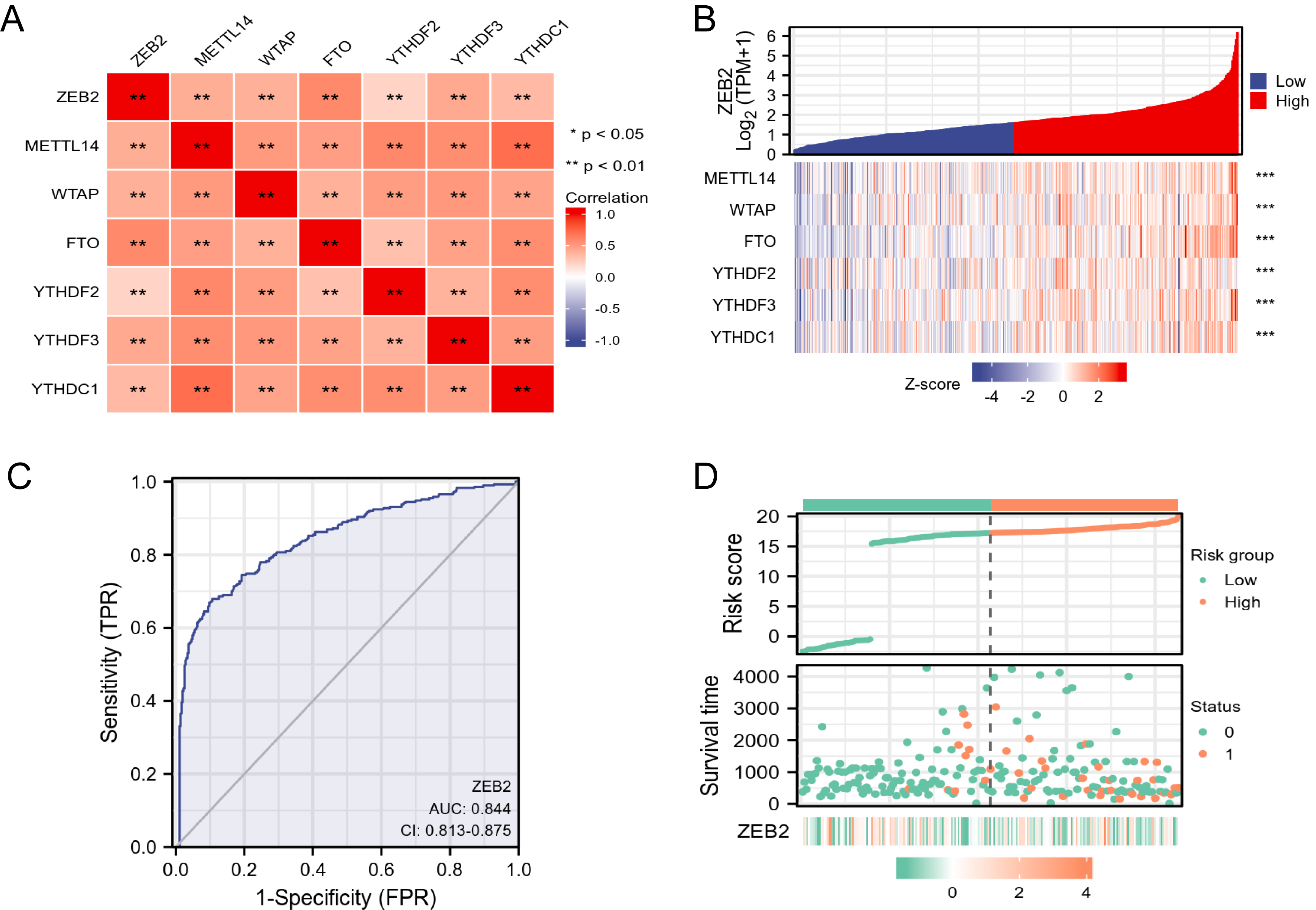

Supplement: Supplementary file 4 [file Image4.TIF]

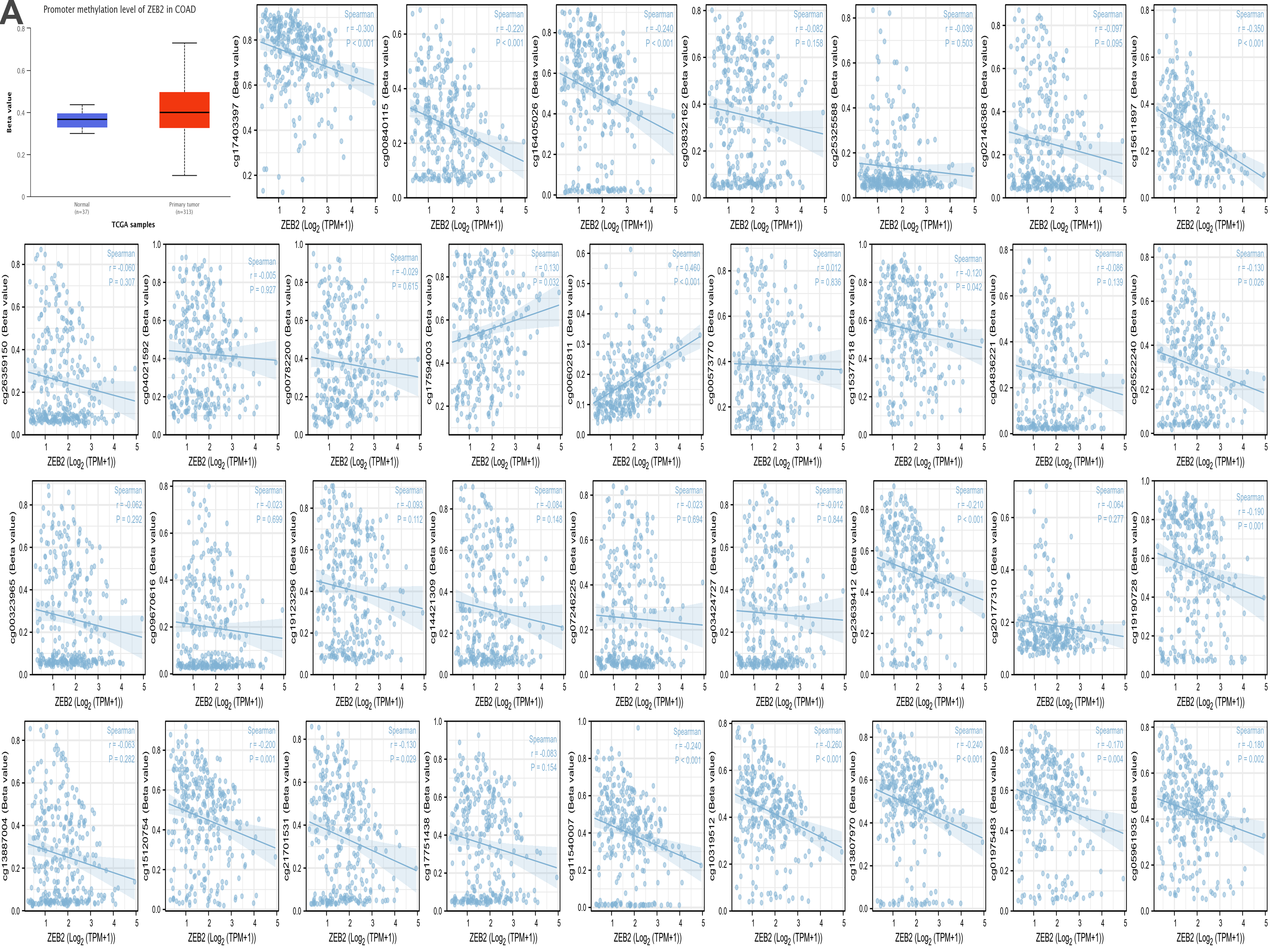

Supplement: Supplementary file 5 [file Image2.TIF]

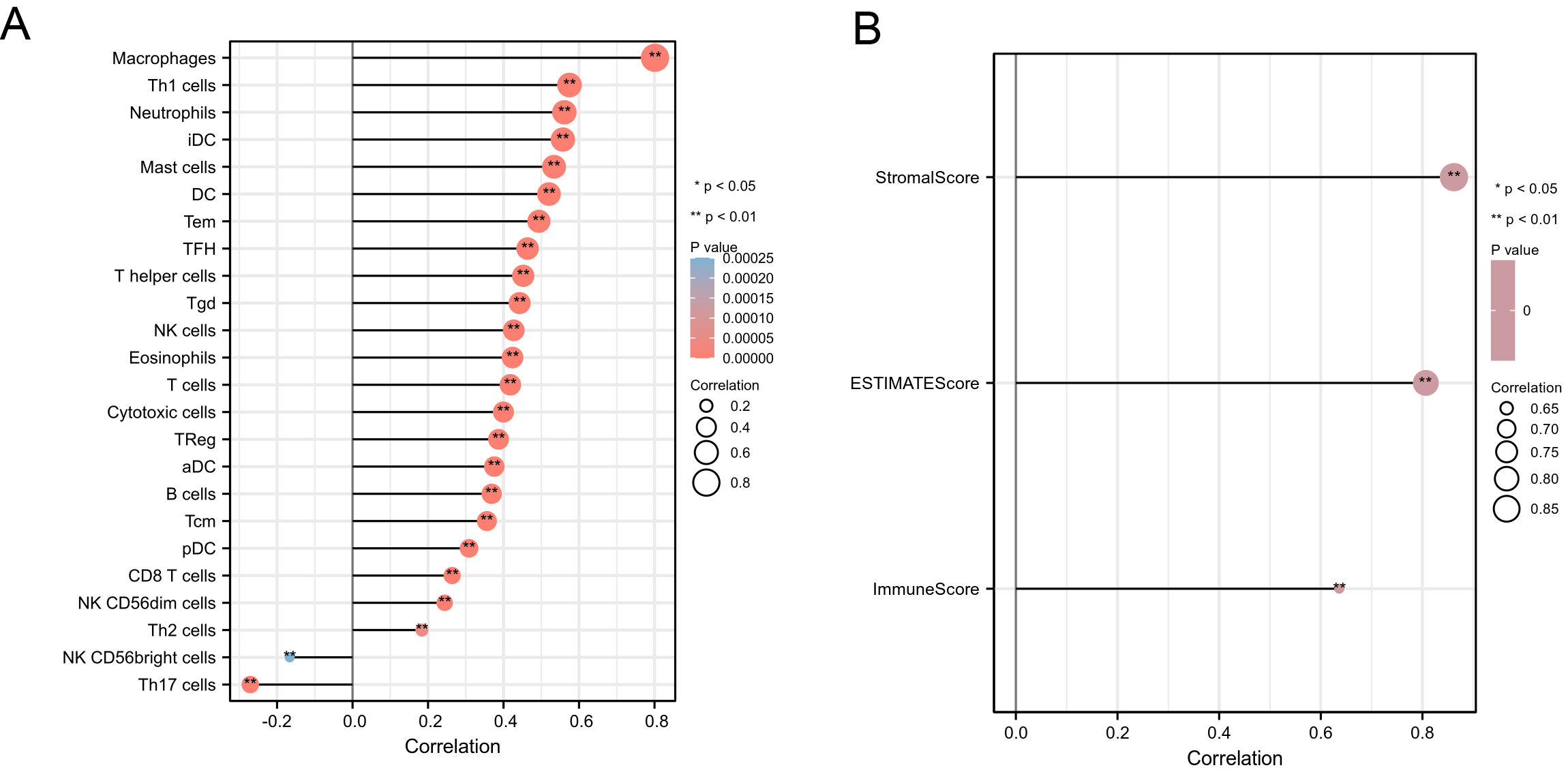

Supplement: Supplementary file 6 [file Image1.TIF]
