# Supplementary material for: Significance of ZEB2 in the immune microenvironment of colon cancer
Source: Front Genet. 2022 Aug 22;13:995333. doi: 10.3389/fgene.2022.995333 (PMC9442042; doi:10.3389/fgene.2022.995333)
Supplement: Supplementary file 2 [file Table1.DOCX]

| Characteristic | Low expression of ZEB2 | High expression of ZEB2 | p |
| --- | --- | --- | --- |
| n | 239 | 239 |  |
| T stage, n (%) |  |  | 0.166 |
| T1 | 7 (1.5%) | 4 (0.8%) |  |
| T2 | 39 (8.2%) | 44 (9.2%) |  |
| T3 | 169 (35.4%) | 154 (32.3%) |  |
| T4 | 23 (4.8%) | 37 (7.8%) |  |
| N stage, n (%) |  |  | 0.804 |
| N0 | 144 (30.1%) | 140 (29.3%) |  |
| N1 | 51 (10.7%) | 57 (11.9%) |  |
| N2 | 44 (9.2%) | 42 (8.8%) |  |
| M stage, n (%) |  |  | 0.271 |
| M0 | 172 (41.4%) | 177 (42.7%) |  |
| M1 | 38 (9.2%) | 28 (6.7%) |  |
| Pathologic stage, n (%) |  |  | 0.511 |
| Stage I | 38 (8.1%) | 43 (9.2%) |  |
| Stage II | 96 (20.6%) | 91 (19.5%) |  |
| Stage III | 63 (13.5%) | 70 (15%) |  |
| Stage IV | 38 (8.1%) | 28 (6%) |  |
| Primary therapy outcome, n (%) |  |  | 0.902 |
| PD | 12 (4.8%) | 13 (5.2%) |  |
| SD | 2 (0.8%) | 2 (0.8%) |  |
| PR | 8 (3.2%) | 5 (2%) |  |
| CR | 104 (41.6%) | 104 (41.6%) |  |
| Gender, n (%) |  |  | 0.169 |
| Female | 105 (22%) | 121 (25.3%) |  |
| Male | 134 (28%) | 118 (24.7%) |  |
| Race, n (%) |  |  | < 0.001 |
| Asian | 7 (2.3%) | 4 (1.3%) |  |
| Black or African American | 41 (13.4%) | 22 (7.2%) |  |
| White | 86 (28.1%) | 146 (47.7%) |  |
| Age, n (%) |  |  | 0.780 |
| <=65 | 95 (19.9%) | 99 (20.7%) |  |
| >65 | 144 (30.1%) | 140 (29.3%) |  |
| Weight, n (%) |  |  | 0.164 |
| <=90 | 78 (28.6%) | 111 (40.7%) |  |
| >90 | 43 (15.8%) | 41 (15%) |  |
| Height, n (%) |  |  | 0.599 |
| <170 | 51 (19.9%) | 76 (29.7%) |  |
| >=170 | 57 (22.3%) | 72 (28.1%) |  |
| BMI, n (%) |  |  | 0.748 |
| <25 | 35 (13.7%) | 52 (20.3%) |  |
| >=25 | 73 (28.5%) | 96 (37.5%) |  |
| Residual tumor, n (%) |  |  | 0.405 |
| R0 | 170 (45.5%) | 176 (47.1%) |  |
| R1 | 1 (0.3%) | 3 (0.8%) |  |
| R2 | 14 (3.7%) | 10 (2.7%) |  |
| CEA level, n (%) |  |  | 0.933 |
| <=5 | 94 (31%) | 102 (33.7%) |  |
| >5 | 50 (16.5%) | 57 (18.8%) |  |
| Perineural invasion, n (%) |  |  | 0.029 |
| NO | 62 (34.3%) | 73 (40.3%) |  |
| YES | 12 (6.6%) | 34 (18.8%) |  |
| Lymphatic invasion, n (%) |  |  | 1.000 |
| NO | 127 (29.3%) | 139 (32%) |  |
| YES | 80 (18.4%) | 88 (20.3%) |  |
| History of colon polyps, n (%) |  |  | 1.000 |
| NO | 131 (32.1%) | 131 (32.1%) |  |
| YES | 73 (17.9%) | 73 (17.9%) |  |
| Colon polyps present, n (%) |  |  | 0.038 |
| NO | 55 (22.1%) | 107 (43%) |  |
| YES | 42 (16.9%) | 45 (18.1%) |  |
| Neoplasm type, n (%) |  |  | 1.000 |
| Colon adenocarcinoma | 239 (50%) | 239 (50%) |  |
| Rectum adenocarcinoma | 0 (0%) | 0 (0%) |  |
| OS event, n (%) |  |  | 0.373 |
| Alive | 183 (38.3%) | 192 (40.2%) |  |
| Dead | 56 (11.7%) | 47 (9.8%) |  |
| PFI event, n (%) |  |  | 1.000 |
| Alive | 175 (36.6%) | 175 (36.6%) |  |
| Dead | 64 (13.4%) | 64 (13.4%) |  |
| DSS event, n (%) |  |  | 1.000 |
| Alive | 192 (41.6%) | 206 (44.6%) |  |
| Dead | 31 (6.7%) | 33 (7.1%) |  |
| Age, meidan (IQR) | 69 (58.5, 76) | 69 (58, 78) | 0.664 |
